# Progress in the Diagnostic and Predictive Evaluation of Crush Syndrome

**DOI:** 10.3390/diagnostics13193034

**Published:** 2023-09-24

**Authors:** Yu Luo, Chunli Liu, Duo Li, Bofan Yang, Jie Shi, Xiaoqin Guo, Haojun Fan, Qi Lv

**Affiliations:** 1 Institution of Disaster and Emergency Medicine, Tianjin University, Tianjin 300072, China; luoyu_2765@tju.edu.cn (Y.L.);; 2 Key Laboratory of Medical Rescue Key Technology and Equipment, Ministry of Emergency Management, Wenzhou 325000, China

**Keywords:** crush syndrome, crush injury, diagnosis, biomarker, predictive evaluation

## Abstract

Crush syndrome (CS), also known as traumatic rhabdomyolysis, is a syndrome with a wide clinical spectrum; it is caused by external compression, which often occurs in earthquakes, wars, and traffic accidents, especially in large-scale disasters. Crush syndrome is the second leading cause of death after direct trauma in earthquakes. A series of clinical complications caused by crush syndrome, including hyperkalemia, myoglobinuria, and, in particular, acute kidney injury (AKI), is the main cause of death in crush syndrome. The early diagnosis of crush syndrome, the correct evaluation of its severity, and accurate predictions of a poor prognosis can provide personalized suggestions for rescuers to carry out early treatments and reduce mortality. This review summarizes various methods for the diagnostic and predictive evaluation of crush syndrome, including urine dipstick tests for a large number of victims, traditional and emerging biomarkers, imaging-assisted diagnostic methods, and developed evaluation models, with the aim of providing materials for scholars in this research field.

## 1. Introduction

Crush injury (CI) refers to direct injury to the body (trunk, limbs, etc.) caused by external compression. Crush syndrome (CS) occurs when the symptoms and signs of CI are not limited to the directly compressed part but show systemic manifestations [1]. In CS, also known as traumatic rhabdomyolysis, injured skeletal muscle cells collapse and release their contents, including myoglobin, creatine kinase, and electrolytes, into the circulation, leading to clinical complications such as myoglobinuria, acute kidney injury (AKI), electrolyte disorders, hypovolemic shock, and multiple-organ dysfunction (MODS) [2,3]. CS affects all vital organs of the body, but the damage to the kidneys is the most prominent, and AKI has become an important factor that threatens the lives of CS patients [4,5,6,7,8]. True CS may require approximately four hours of compression, although this claim has not been systematically investigated [9].

The first mention of rhabdomyolysis in human history is in the Bible, in which quail poisoning was considered to be the cause. German surgeon Frankenthal first reported cases of traumatic rhabdomyolysis and acute kidney injury during the First World War [10]. In 1941, Bywaters and Beall described four patients with crush injuries buried in rubble, with the clinical manifestations of swollen limbs, red-to-brown urine, and shock, and introduced the terminology of ‘crush syndrome’. The four patients died within a week, and the autopsies revealed a brown-pigmented tubular pattern in the renal tubules, similar to that of thrombosis [11,12].

CS often occurs in natural disasters (earthquakes, volcanic eruptions, and landslides) and man-made disasters (wars, traffic accidents, and stampedes), especially in large-scale disasters, which result in heavy property losses and casualties [13]. CS is the second leading cause of death after direct trauma in earthquakes [3,14]. It has been reported that the incidence of CS in earthquake victims is as high as 25%, and the mortality rate can be as high as 21% [15,16]. In addition, CS can be encountered in the emergency room for routine reasons, such as poisoning, stroke, and falling, which can lead to long-term braking, in which the patient’s own body weight becomes the source of compression [17]. Theoretically, any condition that results in prolonged immobility can lead to the development of CS [9].

CS is characterized by systemic involvement, and many of its clinical manifestations are nonspecific. Victims may experience sickness, fever, edema, tachycardia, nausea, vomiting, confusion, anxiety, delirium, tea-colored urine, or anuria [18]. The most common clinical feature is the triad of myalgia, myoglobinuria, and elevated serum muscle enzyme levels; however, the degree and severity of these clinical manifestations vary widely [19]. Therefore, the early diagnosis of CS and the correct evaluation of its severity are crucial for a good prognosis. To realize the early diagnosis of CS and understand the progression of injury, scholars have developed various tests. In this review, we detail the research advances in the diagnosis and assessment of CS to help in the development of more effective evaluation tools in the future.

## 2. Diagnostic Evaluation of Crush Syndrome

### 2.1. Urine Dipstick Test (UDT)

Traumatic rhabdomyolysis is an inexact syndrome with a broad clinical spectrum, and urinalysis can be used as a traditional screening test for rhabdomyolysis. When muscle fibers are damaged by external forces, myoglobin is released during muscle decomposition. The serum myoglobin level increases and reaches the renal threshold, at which point it can be detected in the urine. Orthotoluidine on a urine dipstick (UD) can react with heme molecules in myoglobin and cause hemoglobin molecules to turn blue [20]; therefore, the occurrence of rhabdomyolysis manifests as nonerythrocytic urine and UD positivity. In other words, in the absence of red blood cells in fresh urine, a positive test paper can be used as a surrogate marker for myoglobin. In prehospital settings and emergency conditions, urine paper testing is a highly sensitive and simple screening tool to identify patients at high risk of acute renal failure (ARF) due to crushing, which allows rescuers to perform an initial triage of patients requiring prompt management [21,22].Urine dipstick tests were used in earthquake scenes since as early as the 1995 Kobe earthquake in Japan and the 2003 Bam earthquake in Iran [23,24]. The urine dipstick test is easy to perform and can even be conducted by the patient himself. The patient simply dips the test strip into the urine, compares the color change to the reference label in the instructions, and calls a medical professional for relevant treatment if necessary [25]. However, the lack of means to detect red blood cells under disaster conditions and the presence of blood in the urine may lead to false-positive results. A negative UDT result also cannot completely exclude rhabdomyolysis, and there is a confounding effect of human error in reading dipstick test results. Furthermore, Sameir et al. presented a different view; after retrospectively analyzing the medical records of 228 patients with rhabdomyolysis, they concluded that urine microscopy without red blood cells but with positive urine test paper is not a sensitive screening method for rhabdomyolysis [20]. Although urine is not considered a body fluid, a growing body of research shows the significance of urine as a source of novel biomarkers [26]. A recent study found that urinary tissue inhibitor metal proteinase-2, insulin growth-factor-binding protein-7 (TIMP2*IGFBP7) can be unaffected by pre-existing CKD, enabling the early identification of people at high risk of AKI, especially after cardiac surgery. Based on TIMP2*IGFBP7 levels, AKI can be diagnosed before changes in renal function occur [27]. Due to the ease and non-invasive nature of urine collection, it has significant potential for use in early disease diagnosis in humans. However, due to confounding factors related to human urine, new urine markers face significant challenges in entering clinical practice [28].

### 2.2. Traditional Biomarkers

Currently, the diagnosis of CS relies on a history of injury from prolonged heavy crushing and the results of related laboratory tests. Biomarkers serve as indicators to distinguish between normal and diseased bodily states, and their early detection can improve the success of treatment [29]. Traditional biomarkers for the diagnosis of CS include the following: creatine kinase (CK), serum potassium, myoglobin (Mb), liver enzymes, and bicarbonates, etc.

#### 2.2.1. Creatine Kinase (CK)

Creatine kinase (CK), also known as creatine phosphokinase (CPK), is abundant in skeletal muscle, cardiac muscle, brain tissue, and smooth muscle; it is an important kinase involved in intracellular energy transfer, muscle contraction, and ATP regeneration. The clinical measurement of CK is mainly used for the diagnosis of myocardial diseases and skeletal muscle diseases. As a biomarker of muscle damage, CK is the most sensitive indicator of muscle cell damage and is widely used in the diagnosis of rhabdomyolysis, as well as being an early and sensitive indicator of CS [3]. It is generally accepted that a CK level greater than 1000 IU/L or five times the upper limit of normal (ULN) is a diagnostic indicator of rhabdomyolysis [30,31,32,33]. The normal reference value of CK is affected by the patient’s sex, age, race, and physiological state, so consideration should be comprehensive when making a diagnosis of rhabdomyolysis. Serum CK levels increase at 2–12 h after muscle injury, peaking at 1–3 days, followed by a daily decrease of 39% [34]. Therefore, elevations in CK may be low in patients with delayed admission [35,36]. Furthermore, dynamic changes in CK concentrations are useful in assessing the severity of CS in the early stages [3]. In in-hospital settings, CK testing is the fastest and cheapest means of screening for rhabdomyolysis; however, it cannot be performed at an earthquake site.

Acute kidney injury (AKI) has been extensively studied as one of the few preventable and reversible life-threatening complications of CS [37,38]. Serum CK has been used as the gold standard for predicting acute renal failure. One study found that acute renal failure (ARF) is more likely to occur in patients with higher CK on admission, higher peak CK during admission, and slower serum CK decline [7]. A meta-analysis showed that, in cases of CI, the CK level had an odds ratio (OR) of 14.7 in predicting crush-syndrome-induced acute kidney injury (CS-AKI), but its screening performance was not optimal, and the result needed to be interpreted in conjunction with the results of other risk factors, such as the myoglobin level, urine dipstick test result, uric acid level, and potassium level [39]. Although the CK level is not directly related to the development of AKI, many studies have suggested that a CK level above 5000 IU/L is related to the occurrence of AKI and the need for renal replacement therapy (RRT), as well as a higher risk of complications [33,38,40,41]. Unfortunately, there is no exact clinical threshold at which the CK level indicates the need for RRT, and clinicians often decide whether to perform RRT based on their clinical experience.

#### 2.2.2. Serum Potassium

Disruption of the cell wall leads to the release of potassium and phosphorus into the circulation and an influx of calcium and sodium into cells, resulting in hyperkalemia, hyperphosphatemia, hypocalcemia, and hyponatremia [38,42,43]. In patients with CS following a major earthquake, the immediate life-threatening manifestation is hyperkalemia, which usually occurs within one hour of release. The rapid rise in serum potassium can be reflected in real time on electrocardiography as a shortened QT segment, a high-point T wave, and basal narrowing when the potassium level >5.5 mmol/L; a widened QRS wave group and ST-segment depression when the potassium level is >6.5 mmol/L; and sinus ventricular conduction with sinusoidal waves after QRS-T fusion when the potassium level is >7 mmol/L, which eventually degenerates into ventricular fluttering, ventricular fibrillation, or even cardiac arrest [9]. Therefore, electrocardiographic monitoring of patients with CS is of great significance. Sever et al. investigated and analyzed serum potassium data from victims of the Marmara earthquake in August 1999 and found that admission blood potassium correlated with many clinical and laboratory variables indicating the severity of trauma and was an important predictor of the need for dialysis in victims [44].

#### 2.2.3. Myoglobin (Mb)

Myoglobin (Mb) is a single-chain protein with a relative molecular mass of 16,700 composed of 153 amino acids surrounding a central heme, which is easily excreted in the urine [45]. Tea-colored urine caused by myoglobin is often the first sign of CS and the cause of renal unit damage. Myoglobin is often the first enzyme released in the circulation after any type of muscle injury [46]. Due to the short half-life of myoglobin, its peak level occurs earlier than that of CK, and it is cleared from the circulation within 24 h; it can be used as an early sign of muscle damage to help diagnose rhabdomyolysis. The kidney is the major organ responsible for myoglobin metabolism, and myoglobin-induced nephrotoxicity plays a key role in rhabdomyolysis-related kidney injury through increased oxidative stress, an inflammatory cascade, endothelial dysfunction, immune cell recruitment, vasoconstriction, renal tubular obstruction, and apoptosis [47,48,49,50,51]. In addition, myoglobin nephrotoxicity is exacerbated in the presence of hypovolemia, hypotension, and acidosis [40]. Data from a small-sample prospective cohort study by Virginie et al. suggest that admission myoglobin levels better predict the risk of posttraumatic AKI, allowing the early identification of high-risk patients and the assessment of the severity of rhabdomyolysis [52]. Additionally, it was observed in patients with rhabdomyolysis that the level of myoglobin in the blood was positively correlated with the occurrence of kidney injury and dialysis dependence, which further supports the value of myoglobin as a marker of early kidney injury and suggests the use of high-flow dialysis membranes for early intervention in acute renal injury due to myoglobinuria [53,54].

#### 2.2.4. Liver Enzymes

Abnormal transaminase levels are often considered an indicator of liver damage, and many recent studies have shown that transaminase levels may also indicate rhabdomyolysis. When rhabdomyolysis occurs, significant aspartate aminotransferase (AST) elevations can be detected within 24 h, while the alanine aminotransferase (ALT) level remains in the normal range within 48 h; furthermore, the AST level is often higher than the ALT level, so the AST/ALT ratio is usually greater than 1. Transaminase elevations can be detected over a longer period of time than CK elevations [55,56]. Higher transaminase levels can be observed in patients with severe rhabdomyolysis. Raurich et al. reported that patients with severe rhabdomyolysis with transaminase levels above 1000 IU/L were more likely to develop AKI; additionally, the mortality rate was higher in this population [57]. In a rat CS model, decreased AST and ALT levels were found in the brain [58]. In a study on the serum enzyme profile characteristics of victims of the Wenchuan earthquake in 2008, it was found that the serum CK, lactate dehydrogenase (LDH), AST, ALT, gamma-glutamyl transferase (GGT), and alkaline phosphatase (ALP) levels were all biochemical parameters that could help to estimate the severity of CI and/or CS and prevent the development of further complications [59]. Kathryn et al. found that elevated AST was more common in rhabdomyolysis (present in 93% of patients) and that AST concentrations were highly correlated with CK concentrations [60]. By studying the relationship between AST and CK, Chandel et al. proposed an AST threshold to facilitate the diagnosis and monitoring of rhabdomyolysis in the absence of CK [33]. Lim et al. concluded that ALT, predicted by regression models, biochemical patterns, and trajectories, could serve as a guide in determining the need for more extensive or invasive liver disease testing in patients with rhabdomyolysis; furthermore, ALT could be used as a marker of muscle damage, and reciprocal prediction between peak CK and ALT could be performed [55,61].

Most studies have focused on the renal dysfunction caused by CS, ignoring the damage to liver function. As a syndrome with a broad clinical spectrum, the liver damage associated with rhabdomyolysis cannot be ignored [62,63]. Laitselart et al. found a significant positive linear correlation between CK and ALT, AST, ALP, and bilirubin in patients with rhabdomyolysis due to war injuries and suggested that bilirubin and ALP may be specific markers of rhabdomyolysis-related liver injury. Although the correlation of bilirubin with ALP and CK levels is not as strong as that of transaminases, bilirubin and ALP are more recommended for use in the early identification of liver injury caused by rhabdomyolysis [51,64]. However, because both muscle cells and hepatocytes can produce transaminases, it is difficult to distinguish whether the elevated transaminase levels are due to rhabdomyolysis, liver injury, or both, in the presence of rhabdomyolysis [51]. Therefore, for patients with abnormal liver enzyme levels, the possibility of CS should be considered in combination with the relevant medical history, along with the possibility of liver damage due to CS.

#### 2.2.5. Bicarbonate

Bicarbonate is a commonly used monitoring indicator in blood gas analysis. Muckart et al. conducted a prospective study of 64 patients with soft tissue injury and found that an initial venous bicarbonate concentration (VBC) lower than 17 mmol/L could predict the occurrence of myoglobin-induced acute renal failure [65]. This finding was confirmed by Skinner et al. using intravenous bicarbonate as a reliable tool for risk stratification in patients with crush injuries [66]. In another retrospective study, it was found that intravenous bicarbonate was more powerful than CK in predicting the risk of renal failure in patients with CS. Considering the ease and low cost of bicarbonate testing, intravenous bicarbonate measurement is recommended for patients with crush injuries, while CK measurement is considered a complementary modality [67].

### 2.3. Non-Traditional Biomarkers

With the development of multiomics, some relatively innovative, “non-traditional” biomarkers have also been proposed recently for the early diagnosis and evaluation of the occurrence and development of CS.

#### 2.3.1. Neutrophil-Gelatinase-Associated Lipocalin (NGAL)

Neutrophil-gelatinase-associated lipocalin (NGAL), also known as lipocalin-2 (LCN2), siderocalin, or 24p3, is a member of the lipocalin family [68,69]. NGAL is a 25-kDa, small-molecular-weight, secreted protein that was initially identified in activated neutrophils but has since been described in many other cell types, including kidney cells, endothelial cells, liver cells, smooth muscle cells, cardiomyocytes, neurons, and various immune cell populations [68,70]. NGAL has powerful functions that are related to inflammation, embryonic development, immune responses, chemotaxis, signal transduction, differentiation and proliferation, and tumorigenesis and development, in addition to the function of transporting small hydrophobic molecules [71]. In the last decade, NGAL has received much attention from nephrologists as a noninvasive early biomarker of AKI. After acute kidney injury, NGAL is synthesized and secreted by the thick ascending limb of Henle’s loop and the collecting duct and becomes a sensitive and specific biomarker of kidney injury detectable in urine and blood [72,73]. Traditional kidney injury markers such as serum creatinine (Scr) and blood urea nitrogen (BUN) are delayed and unreliable indicators of AKI and are only relevant when there is a substantial loss of kidney function [70,74,75]. Although NGAL is not a direct diagnostic indicator of CS, early monitoring of NGAL levels can detect crush-related AKI in good time. Elevated NGAL levels have been reported in two patients with CS in mudslide disasters as a good predictor of acute kidney injury and as a precursor to increased serum creatinine levels [8].

#### 2.3.2. Alpha-1-Acid Glycoprotein (a1-AGP)

Alpha-1-acid glycoprotein (a1-AGP) is a nonspecific acute-phase protein with inflammatory and immunomodulatory properties that is mainly synthesized by the liver [76,77]. In mouse experiments, a1-AGP has been shown to protect against ischemia–reperfusion injury through anti-apoptotic and anti-inflammatory effects [77,78]. We previously used isobaric tags for relative and absolute quantitation (iTRAQ) combined with liquid chromatography–tandem mass spectrometry (LC–MS/MS) to identify serum biomarkers in CS rats. Our findings suggest that a1-AGP is a nonnegligible biomarker in CS and is of great significance not only in predicting the severity of CS but also as a mediator of CS-induced AKI; however, its use as a target in the treatment of CS-induced AKI requires detailed mechanistic studies in future work [3].

#### 2.3.3. MicroRNA (miRNA)

Circulating microRNAs (miRNAs), newly discovered small noncoding RNAs that regulate protein levels through transcription, are expected to constitute unique accessible biomarkers for detecting tissue injury due to their size, abundance, tissue specificity, and relative stability in plasma [79]. In rat experiments, Laterza et al. observed increased plasma concentrations of miR-122 and miR-133a, which could correspond to liver and muscle damage, respectively [80]. Bailey et al. performed a comprehensive assessment of liver- and skeletal-muscle-specific miRNAs and found that miR-122 and miR-192 in liver and miR-1, miR-133a, miR-133b and miR-206 in skeletal muscle all outperformed ALT and AST as traditional liver injury biomarkers and CK as a skeletal muscle injury marker [81], allowing for the detection of different miRNAs to differentiate between muscle and liver injury. This indicates their potential as useful diagnostic biomarkers.

### 2.4. Imaging Methods

Ultrasound (US), computed tomography (CT), and magnetic resonance imaging (MRI) can confirm and support the diagnosis of rhabdomyolysis.

#### 2.4.1. Ultrasound (US)

Ultrasound (US) is a nonradiative, simple, rapid examination that can feasibly be performed at the bedside and is often used in the diagnosis of acute muscular lesions [82]. After the occurrence of disasters such as earthquakes, US is a relatively practical examination method. US can potentially be used to detect patients without clinical symptoms or signs and achieve an early diagnosis [83,84]. US can clearly show the extent of muscle lesions and the presence of internal fluid accumulation in patients with rhabdomyolysis caused by compression. Ultrasound-guided aspiration can be performed in cases where there is a large amount of fluid accumulation in muscle tissue. When the etiology is unknown, ultrasound-guided muscle puncture can also be used as a pathological examination [84]. Compared with other imaging modalities, US is more suitable for follow-up examinations to track the effect of treatment. After the Wenchuan earthquake in 2008, conventional ultrasound was used to diagnose rhabdomyolysis and acute osteofascial compartment syndrome (AOCS) due to compression [84]. Zhang C-D et al. constructed a skeletal muscle CI model using a balloon cuff to compress the hind limbs of rabbits and found that contrast-enhanced ultrasound (CEUS) was more sensitive than conventional ultrasound in identifying the initial microcirculatory changes in crushed muscles; thus, it may play an important role in the early diagnosis of muscle crush injuries [82]. In addition, a recent study by Zhao et al. demonstrated that CEUS is a sensitive tool for assessing renal perfusion changes in rhabdomyolysis-induced acute kidney injury [85].

#### 2.4.2. Computed Tomography (CT)

Muscles with rhabdomyolysis often show focal shadows of hypodensity on CT, but this feature is often nonspecific. Similar signs can be found in cases of suppurative myositis, abscesses, and tumors [86]. However, Russ et al. found that CT images in four of eight patients with rhabdomyolysis showed areas of abnormally high density, consistent with skeletal muscle calcification; this is the opposite of the conventional CT presentation and could help to diagnose occult rhabdomyolysis [87]. Similarly, in the 1995 Kobe earthquake, Nakanishi, K. et al. observed muscle calcification shadows on CT images of five patients with crush injuries, three of whom showed a decrease in calcification over time [88].

#### 2.4.3. Magnetic Resonance Imaging (MRI)

Magnetic resonance imaging (MRI), as an imaging tool with good soft tissue contrast, is the method of choice for assessing the extent and distribution of rhabdomyolysis. Many scholars have compared the performance of ultrasound, CT, and MRI in evaluating rhabdomyolysis and found that the sensitivity of MRI in detecting abnormal muscles was higher than that of CT and ultrasound (100%, 62%, and 42%, respectively) [18,36,86,89]. The affected muscles show an increased signal intensity on T2-weighted spin echo imaging and a decreased signal intensity on T1-weighted imaging. In the acute phase, the abnormal signal is associated with an increase in the cross-sectional diameter of the affected muscle [18,88,89,90]. Zhang, L. et al. suggested that the combined application of MRI and magnetic resonance angiography (MRA) to locate the affected muscles and distinguish them from unaffected muscles would be more helpful in assessing the need for surgical treatment [89]. In addition, Chia-Hung et al. retrospectively analyzed the MRI and CT images of ten patients with rhabdomyolysis and distinguished two different types based on the imaging, which could help to identify rhabdomyolysis with different etiologies [35].

Although imaging methods for the diagnosis of rhabdomyolysis are not specific and often require a combination of clinical and laboratory data, radiographic images provide information that can help assess the extent and distribution of rhabdomyolysis. Osteofascial compartment syndrome (OCS) is a common complication of CS. Radiological techniques, especially MRI, allow for the precise identification of the affected muscles and subsequent targeted decompression therapy. In addition, near-infrared spectroscopy (NIRS) can be used to detect OCS by measuring tissue oxygenation [91]. In conclusion, the use of radiographic techniques is important to localize diseased muscles and differentiate them from nondiseased muscles.

## 3. Predictive Evaluation of Crush Syndrome

Due to the complexity of the pathophysiology of CS, avoidance of fatal CS depends on the early diagnosis and timely management of high-risk patients [24,92]. Therefore, scholars have developed scoring systems to assess the risk of adverse outcomes in patients with rhabdomyolysis by modeling screening metrics. In 2007, Aoki et al. retrospectively analyzed CI clinical data from the Kobe earthquake and used data mining methods to develop two types of predictive triage models—an initial evaluation for use in the field and a secondary assessment for use at the hospital—to help nonmedical rescuers distinguish between patients with CS of different severities and make better use of the limited medical resources available after disasters. The initial triage model included the pulse rate, delayed rescue, and abnormal urine color, with an AUC of 0.73; the secondary model included the white blood cell (WBC) count, tachycardia, abnormal urine color, and hyperkalemia, with an AUC of 0.76 [92]. AKI has received widespread attention as one of the most serious and reversible complications of CS [93]. Renal disease due to CS presents asymptomatically in the early stages, and, without intervention, most renal function will later be lost. Therefore, the early screening of patients at risk for AKI after crush-induced rhabdomyolysis is critical. In 2008, Najafi et al. developed two scoring models (the “rule of thumb” and the AKI index) based on biochemical data from Bam earthquake victims to differentiate between those at high and nonhigh risk of AKI in order to provide early prophylactic hydration therapy for those at high risk of AKI and reduce mortality [94]. In 2009, a prospective study by Paul et al. found a higher risk of ARF in patients with lower-extremity crush injuries and reported the potential value of the Mangled Extremity Severity Score (MESS) in predicting the incidence of AKI in patients with crush injuries [95]. In 2013, the McMahon score, consisting of initial creatinine, calcium, CPK, phosphate, and bicarbonate parameters was developed to predict the probability of renal replacement therapy (RRT) or in-hospital death in patients with rhabdomyolysis [96]. These predictive tools use fewer test parameters to identify high-risk rhabdomyolysis patients in need of early intervention; they can help in the classification of injuries in high-volume CS patients at disaster scenes, help rescuers in decision planning, and optimize medical resource allocation. However, the greatest shortcoming of these predictive models lies in the limitations of retrospective studies. In addition, most scoring systems, except for the McMahon scoring system, lack external data validation.

## 4. Conclusions and Prospects

**Figure 1:**
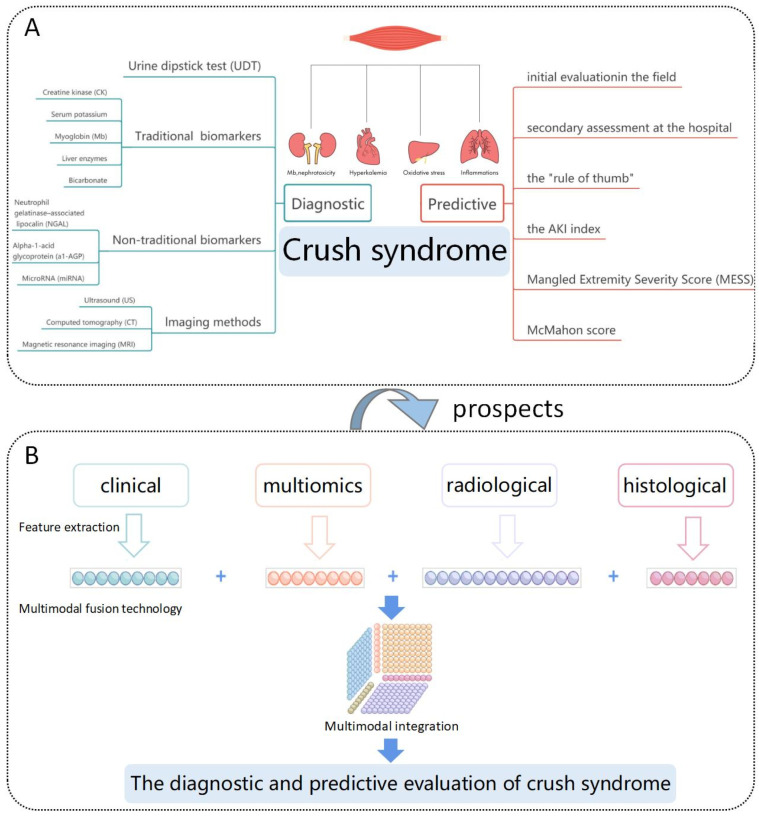
(**A**) Various diagnostic and predictive evaluation methods described in this paper. (**B**) Future prospects for the evaluation of CS using multimodal fusion technology. Feature extraction is carried out for each single data mode, and multimodal fusion technology is used to integrate different modes of data to realize the diagnostic and predictive evaluation of CS.

Rhabdomyolysis is the core component of CS, and the toxic effects of muscle cell contents can involve multiple organs, such as the kidneys, heart, and lungs, leading to a series of complications, such as acute kidney injury (AKI), hypovolemic shock, hyperkalemia, acute respiratory distress syndrome (ARDS), and diffuse intravascular coagulation (DIC) [43]. Since CS involves more than just muscles, systemic effects on organs also occur. When assessing biomarker levels, it is important to pay attention not only to muscle damage but also to the various complications associated with CS. The in-hospital diagnosis of CS has long been a nonissue; however, the question of how to rapidly identify high-risk patients in the face of large volumes of casualties in a chaotic prehospital setting still needs to be addressed. This review summarizes the common screening methods, conventional and emerging biomarkers, diagnostic imaging tools, and various predictive scoring systems for CS. Other than NGAL, which has been promoted and applied in some hospitals, other potential markers, such as a1-AGP and miRNAs, are still in the preclinical research stage and have not been applied in clinical practice; however, they may lead to the development of attractive diagnostic tools in the future. Currently, the diagnosis of CS cannot be achieved by a single test, and the various scoring systems that have been developed also have many shortcomings (Table 1 and Figure 1A).

With the advancement and application of multimodal technology in the medical field, integrating multiple data modalities to refine disease management has attracted widespread attention. Examples include integrating contrast-enhanced computed tomography and clinical features for the differential diagnosis of hepatocellular carcinoma [97], integrating multiomics analysis for predicting the severity of COVID-19 [98], and integrating clinical data, mRNA expression data, microRNA expression data, and histopathology images for pancancer prognosis predictions [99]. Multimodal integration technology shows significant advantages in describing the occurrence, development, and prognosis of diseases. Therefore, we forecast that there will be great potential for the early diagnosis and assessment of CS in the future based on medical data from multiple modalities (including clinical data, multiomics data, radiological data, and histological data) through multimodal fusion technology (Figure 1B).

## Figures and Tables

**Table 1:** Characteristics of diagnostic evaluation methods for crush syndrome.

Categories	Diagnostic Evaluation	Characteristics
Traditional Biomarkers	UDT	Screening test, easy to operate [23,24,25]
	CK	Easy, sensitive [3,34]
	Serum potassium	Combined ECG to monitor cardiac function [9]
	Mb	Cause of renal unit damage, early sign of muscle damage [46]
	Liver enzymes	Indicator of liver injury, elevated AST more common [55,56]
	Bicarbonate	Predict the risk of renal failure in CS patients [65]
Non-Traditional Biomarkers	NGAL	A noninvasive, early biomarker of AKI and already in clinical application [72,73]
	a1-AGP	Predict the severity of CS (animal studies) [3]
	miRNA	Distinguish between muscle damage and liver damage (animal studies) [80,81]
Imaging Methods	US	Simple, fast, and bedside accessible [82,83,84]
	CT	Detect occult rhabdomyolysis [87]
	MRI	Good soft tissue contrast, higher sensitivity than US and CT [18,36,86,89]

Abbreviations: CS, crush syndrome; UDT, urine dipstick test; CK, creatine kinase; ECG, electrocardiogram; Mb, myoglobin; AST, aspartate aminotransferase; NGAL, neutrophil gelatinase–associated lipocalin; AKI, acute kidney injury; a1-AGP, alpha-1-acid glycoprotein; miRNA, MicroRNA; US, ultrasound; CT, computed tomography; MRI, magnetic resonance imaging.

## Data Availability

Data sharing is not applicable to this article as no new data were created or analyzed in this study.

## References

[1] Smith J., Greaves I. (2003). Crush injury and crush syndrome: A review. J. Trauma.

[2] Kodadek L., Carmichael S.P., Seshadri A., Pathak A., Hoth J., Appelbaum R., Michetti C.P., Gonzalez R.P. (2022). Rhabdomyolysis: An American Association for the Surgery of Trauma Critical Care Committee Clinical Consensus Document. Trauma Surg. Acute Care Open.

[3] Lv Q., Long M., Wang X., Shi J., Wang P., Guo X., Song J., Midgley A.C., Fan H., Hou S. (2021). The Role of Alpha-1-Acid Glycoprotein in the Diagnosis and Treatment of Crush Syndrome-Induced Acute Kidney Injury. Shock.

[4] Qiao O., Wang X., Wang Y., Li N., Gong Y. (2023). Ferroptosis in acute kidney injury following crush syndrome: A novel target for treatment. J. Adv. Res..

[5] He Q., Wang F., Li G., Chen X., Liao C., Zou Y., Zhang Y., Kang Z., Yang X., Wang L. (2011). Crush syndrome and acute kidney injury in the Wenchuan Earthquake. J. Trauma.

[6] Okubo K., Kurosawa M., Kamiya M., Urano Y., Suzuki A., Yamamoto K., Hase K., Homma K., Sasaki J., Miyauchi H. (2018). Macrophage extracellular trap formation promoted by platelet activation is a key mediator of rhabdomyolysis-induced acute kidney injury. Nat. Med..

[7] De Meijer A.R., Fikkers B.G., de Keijzer M.H., van Engelen B.G.M., Drenth J.P.H. (2003). Serum creatine kinase as predictor of clinical course in rhabdomyolysis: A 5-year intensive care survey. Intensive Care Med..

[8] Donato V., Noto A., Lacquaniti A., Bolignano D., Versaci A., David A., Spinelli F., Buemi M. (2011). Levels of neutrophil gelatinase-associated lipocalin in 2 patients with crush syndrome after a mudslide. Am. J. Crit. Care.

[9] Burns K., Cone D.C., Portereiko J.V. (2010). Complex extrication and crush injury. Prehospital Emerg. Care.

[10] Aleckovic-Halilovic M., Pjanic M., Mesic E., Storrar J., Woywodt A. (2021). From quail to earthquakes and human conflict: A historical perspective of rhabdomyolysis. Clin. Kidney J..

[11] Bywaters E.G.L., Beall D. (1941). Crush Injuries with Impairment of Renal Function. BMJ.

[12] Peiris D. (2017). A historical perspective on crush syndrome: The clinical application of its pathogenesis, established by the study of wartime crush injuries. J. Clin. Pathol..

[13] Sever M.S., Lameire N., van Biesen W., Vanholder R. (2015). Disaster nephrology: A new concept for an old problem. Clin. Kidney J..

[14] Sever M.S., Vanholder R. (2013). Management of crush victims in mass disasters: Highlights from recently published recommendations. Clin. J. Am. Soc. Nephrol..

[15] Bartal C., Zeller L., Miskin I., Sebbag G., Karp E., Grossman A., Engel A., Carter D., Kreiss Y. (2011). Crush Syndrome: Saving More Lives in Disasters. Arch. Intern. Med..

[16] Ersoy A., Yavuz M., Usta M., Ercan I., Aslanhan I., Güllülü M., Kurt E., Emir G., Dilek K., Yurtkuran M. (2003). Survival analysis of the factors affecting in mortality in injured patients requiring dialysis due to acute renal failure during the Marmara earthquake: Survivors vs non-survivors. Clin. Nephrol..

[17] Greaves I., Porter K., Smith J.E. (2003). Consensus statement on the early management of crush injury and prevention of crush syndrome. J. R. Army Med. Corp..

[18] Moratalla M.B., Braun P., Fornas G.M. (2008). Importance of MRI in the diagnosis and treatment of rhabdomyolysis. Eur. J. Radiol..

[19] Long S., Garrett J., Bhargava P., Aguilar G., Simoncini A., Sangster G. (2017). Multimodality imaging findings in rhabdomyolysis and a brief review of differential diagnoses. Emerg. Radiol..

[20] Alhadi S.A., Ruegner R., Snowden B., Hendey G.W. (2014). Urinalysis is an inadequate screen for rhabdomyolysis. Am. J. Emerg. Med..

[21] Young S.E., Miller M.A., Docherty M. (2009). Urine dipstick testing to rule out rhabdomyolysis in patients with suspected heat injury. Am. J. Emerg. Med..

[22] Alavi-Moghaddam M., Safari S., Najafi I., Hosseini M. (2012). Accuracy of urine dipstick in the detection of patients at risk for crush-induced rhabdomyolysis and acute kidney injury. Eur. J. Emerg. Med..

[23] Oda Y., Shindoh M., Yukioka H., Nishi S., Fujumori M., Asada A. (1997). Crush Syndrome Sustained in the 1995 Kobe, Japan, Earthquake; Treatment and Outcome. Ann. Emerg. Med..

[24] Amini M., Sharifi A., Najafi I., Eghtesadi-Araghi P., Rasouli M.R. (2009). Role of dipstick in detection of haeme pigment due to rhabdomyolysis in victims of Bam earthquake. EMHJ-East. Mediterr. Health J..

[25] Safari S., Yousefifard M., Hashemi B., Baratloo A., Forouzanfar M.M., Rahmati F., Motamedi M., Najafi I. (2016). The Role of Scoring Systems and Urine Dipstick in Prediction of Rhabdomyolysis-induced Acute Kidney Injury. Iran. J. Kidney Dis..

[26] Gao Y. (2013). Urine-an untapped goldmine for biomarker discovery?. Sci. China Life Sci..

[27] Lacquaniti A., Ceresa F., Campo S., Barbera G., Caruso D., Palazzo E., Patanè F., Monardo P. (2023). Acute Kidney Injury and Sepsis after Cardiac Surgery: The Roles of Tissue Inhibitor Metalloproteinase-2, Insulin-like Growth Factor Binding Protein-7, and Mid-Regional Pro-Adrenomedullin. J. Clin. Med..

[28] Wei J., Gao Y. (2021). Early disease biomarkers can be found using animal models urine proteomics. Expert Rev. Proteom..

[29] Suntornsuk W., Suntornsuk L. (2020). Recent applications of paper-based point-of-care devices for biomarker detection. Electrophoresis.

[30] O’Carroll C., Fenwick R. (2020). Rhabdomyolysis: A case-based critical reflection on its causes and diagnosis. Emerg. Nurse.

[31] Long B., Koyfman A., Gottlieb M. (2019). An evidence-based narrative review of the emergency department evaluation and management of rhabdomyolysis. Am. J. Emerg. Med..

[32] Chavez L.O., Leon M., Einav S., Varon J. (2016). Beyond muscle destruction: A systematic review of rhabdomyolysis for clinical practice. Crit. Care.

[33] Chandel A., Brusher K., Hall V., Howard R.S., Clark P.A. (2019). Diagnosis and Management of Rhabdomyolysis in the Absence of Creatine Phosphokinase: A Medical Record Review. Mil. Med..

[34] Nance J.R., Mammen A.L. (2015). Diagnostic evaluation of rhabdomyolysis. Muscle Nerve.

[35] Lu C.-H., Tsang Y.-M., Yu C.-W., Wu M.-Z., Hsu C.-Y., Shih T.T.-F. (2007). Rhabdomyolysis: Magnetic Resonance Imaging and Computed Tomography Findings. J. Comput. Assist. Tomogr..

[36] Nakahara K., Tanaka H., Masutani K., Yanagida T., Kashiwagi M., Mizumasa T., Masuda K., Hirakata H., Fujishima M. (1999). The value of computed tomography and magnetic resonance imaging to diagnose rhabdomyolysis in acute renal failure. Nephrol. Dial. Transplant..

[37] Sever M.S., Vanholder R., Lameire N. (2006). Management of crush-related injuries after disasters. N. Engl. J. Med..

[38] Genthon A., Wilcox S.R. (2014). Crush syndrome: A case report and review of the literature. J. Emerg. Med..

[39] Safari S., Yousefifard M., Hashemi B., Baratloo A., Forouzanfar M.M., Rahmati F., Motamedi M., Najafi I. (2016). The value of serum creatine kinase in predicting the risk of rhabdomyolysis-induced acute kidney injury: A systematic review and meta-analysis. Clin. Exp. Nephrol..

[40] Simpson J.P., Taylor A., Sudhan N., Menon D.K., Lavinio A. (2016). Rhabdomyolysis and acute kidney injury: Creatine kinase as a prognostic marker and validation of the McMahon Score in a 10-year cohort: A retrospective observational evaluation. Eur. J. Anaesthesiol..

[41] Yeh Y.-C., Chen C.-C., Lin S.-H. (2022). Case report: Severe rhabdomyolysis and acute liver injury in a high-altitude mountain climber. Front. Med..

[42] Safari S., Eshaghzade M., Najafi I., Baratloo A., Hashemi B., Forouzanfar M.M., Rahmati F. (2017). Trends of Serum Electrolyte Changes in Crush syndrome patients of Bam Earthquake; a Cross sectional Study. Emergency.

[43] Gonzalez D. (2005). Crush syndrome. Crit. Care Med..

[44] Sever M.S., Erek E., Vanholder R., Kantarci G., Yavuz M., Turkmen A., Ergin H., Tulbek M.Y., Duranay M., Manga G. (2003). Serum potassium in the crush syndrome victims of the Marmara disaster. Clin. Nephrol..

[45] Takano T. (1977). Structure of myoglobin refined at 2-0 A resolution. II. Structure of deoxymyoglobin from sperm whale. J. Mol. Biol..

[46] Nilsson A., Ibounig T., Lyth J., Alkner B., von Walden F., Fornander L., Rämö L., Schmidt A., Schilcher J. (2022). BioFACTS: Biomarkers of rhabdomyolysis in the diagnosis of acute compartment syndrome—Protocol for a prospective multinational, multicentre study involving patients with tibial fractures. BMJ Open.

[47] Samuel H.U., Balasubramaniyan T., Thirumavalavan S., Vasudevan C., Senthil Kumar R.P., Murugesan V., Abraham A. (2021). Rhabdomyolysis with myoglobin-induced acute kidney injury: A case series of four cases. Indian J. Pathol. Microbiol..

[48] Panizo N., Rubio-Navarro A., Amaro-Villalobos J.M., Egido J., Moreno J.A. (2015). Molecular Mechanisms and Novel Therapeutic Approaches to Rhabdomyolysis-Induced Acute Kidney Injury. Kidney Blood Press. Res..

[49] Zorova L.D., Pevzner I.B., Chupyrkina A.A., Zorov S.D., Silachev D.N., Plotnikov E.Y., Zorov D.B. (2016). The role of myoglobin degradation in nephrotoxicity after rhabdomyolysis. Chem. Biol. Interact..

[50] Plotnikov E.Y., Chupyrkina A.A., Pevzner I.B., Isaev N.K., Zorov D.B. (2009). Myoglobin causes oxidative stress, increase of NO production and dysfunction of kidney’s mitochondria. Biochim. Biophys. Acta.

[51] Laitselart P., Derely J., Daban J.-L., de Rudnicki S., Libert N. (2022). Relationship between creatine kinase and liver enzymes in war wounded with rhabdomyolysis. Injury.

[52] Tarazona V., Figueiredo S., Hamada S., Pochard J., Haines R.W., Prowle J.R., Duranteau J., Vigué B., Harrois A. (2021). Admission serum myoglobin and the development of acute kidney injury after major trauma. Ann. Intensive Care.

[53] Premru V., Kovač J., Ponikvar R. (2013). Use of myoglobin as a marker and predictor in myoglobinuric acute kidney injury. Ther. Apher. Dial..

[54] Zhang L., Kang Y., Fu P., Cao Y., Shi Y., Liu F., Hu Z., Su B., Tang W., Qin W. (2012). Myoglobin clearance by continuous venous-venous haemofiltration in rhabdomyolysis with acute kidney injury: A case series. Injury.

[55] Lim A.K. (2020). Abnormal liver function tests associated with severe rhabdomyolysis. World J. Gastroenterol..

[56] Radke J.B., Algren D.A., Chenoweth J.A., Owen K.P., Ford J.B., Albertson T.E., Sutter M.E. (2018). Transaminase and Creatine Kinase Ratios for Differentiating Delayed Acetaminophen Overdose from Rhabdomyolysis. West. J. Emerg. Med..

[57] Raurich J.M., Llompart-Pou J.A., Rodríguez-Yago M., Ferreruela M., Royo C., Ayestarán I. (2015). Role of Elevated Aminotransferases in ICU Patients with Rhabdomyolysis. Am. Surg..

[58] Desai S.N., Desai P.V. (2008). Aspartate aminotransferase and alanine aminotransferase activities of rat brain during crush syndrome. Neurosci. Lett..

[59] Feng J., Zen P., Liu Y., Luo J., Jiang W., Peng G. (2009). Serum enzyme profile characteristics of victims following the Wenchuan earthquake in China. Clin. Chem. Lab. Med..

[60] Weibrecht K., Dayno M., Darling C., Bird S.B. (2010). Liver aminotransferases are elevated with rhabdomyolysis in the absence of significant liver injury. J. Med. Toxicol..

[61] Lim A.K.H., Arumugananthan C., Lau Hing Yim C., Jellie L.J., Wong E.W.W., Junckerstorff R.K. (2019). A Cross-Sectional Study of the Relationship between Serum Creatine Kinase and Liver Biochemistry in Patients with Rhabdomyolysis. J. Clin. Med..

[62] Akmal M., Massry S.G. (1990). Reversible hepatic dysfunction associated with rhabdomyolysis. Am. J. Nephrol..

[63] Fidler S., Fagan E., Williams R., Dewhurst I., Cory C.E. (1988). Heatstroke and rhabdomyolysis presenting as fulminant hepatic failure. Postgrad. Med. J..

[64] Dufour D.R., Lott J.A., Nolte F.S., Gretch D.R., Koff R.S., Seeff L.B. (2000). Diagnosis and monitoring of hepatic injury. II. Recommendations for use of laboratory tests in screening, diagnosis, and monitoring. Clin. Chem..

[65] Muckart D.J., Moodley M., Naidu A.G., Reddy A.D., Meineke K.R. (1992). Prediction of acute renal failure following soft-tissue injury using the venous bicarbonate concentration. J. Trauma.

[66] Skinner D.L., Laing G.L., Bruce J., Biccard B., Muckart D.J.J. (2017). Validating the utilisation of venous bicarbonate as a predictor of acute kidney injury in crush syndrome from sjambok injuries. S. Afr. Med. J..

[67] Buitendag J.J.P., Patel M.Q., Variawa S., Fichardt J., Mostert B., Goliath A., Clarke D.L., Oosthuizen G.V. (2021). Venous bicarbonate and creatine kinase as diagnostic and prognostic tools in the setting of acute traumatic rhabdomyolysis. S. Afr. Med. J..

[68] Schreiber A., Rousselle A., Klocke J., Bachmann S., Popovic S., Bontscho J., Schmidt-Ott K.M., Siffrin V., Jerke U., Ashraf M.I. (2020). Neutrophil Gelatinase-Associated Lipocalin Protects from ANCA-Induced GN by Inhibiting TH17 Immunity. J. Am. Soc. Nephrol..

[69] Haase M., Bellomo R., Devarajan P., Schlattmann P., Haase-Fielitz A. (2009). Accuracy of neutrophil gelatinase-associated lipocalin (NGAL) in diagnosis and prognosis in acute kidney injury: A systematic review and meta-analysis. Am. J. Kidney Dis..

[70] Buonafine M., Martinez-Martinez E., Jaisser F. (2018). More than a simple biomarker: The role of NGAL in cardiovascular and renal diseases. Clin. Sci..

[71] Bolignano D., Coppolino G., Lacquaniti A., Nicocia G., Buemi M. (2008). Pathological and prognostic value of urinary neutrophil gelatinase-associated lipocalin in macroproteinuric patients with worsening renal function. Kidney Blood Press. Res..

[72] Paragas N., Qiu A., Zhang Q., Samstein B., Deng S.-X., Schmidt-Ott K.M., Viltard M., Yu W., Forster C.S., Gong G. (2011). The Ngal reporter mouse detects the response of the kidney to injury in real time. Nat. Med..

[73] Mishra J., Ma Q., Prada A., Mitsnefes M., Zahedi K., Yang J., Barasch J., Devarajan P. (2003). Identification of neutrophil gelatinase-associated lipocalin as a novel early urinary biomarker for ischemic renal injury. J. Am. Soc. Nephrol..

[74] Clerico A., Galli C., Fortunato A., Ronco C. (2012). Neutrophil gelatinase-associated lipocalin (NGAL) as biomarker of acute kidney injury: A review of the laboratory characteristics and clinical evidences. Clin. Chem. Lab. Med..

[75] Devarajan P. (2010). Review: Neutrophil gelatinase-associated lipocalin: A troponin-like biomarker for human acute kidney injury. Nephrology.

[76] Hochepied T., Berger F.G., Baumann H., Libert C. (2003). α1-Acid glycoprotein: An acute phase protein with inflammatory and immunomodulating properties. Cytokine Growth Factor Rev..

[77] Daemen M.A., Heemskerk V.H., van’t Veer C., Denecker G., Wolfs T.G., Vandenabeele P., Buurman W.A. (2000). Functional protection by acute phase proteins alpha(1)-acid glycoprotein and alpha(1)-antitrypsin against ischemia/reperfusion injury by preventing apoptosis and inflammation. Circulation.

[78] De Vries B., Walter S.J., Wolfs T.G.A.M., Hochepied T., Räbinä J., Heeringa P., Parkkinen J., Libert C., Buurman W.A. (2004). Exogenous alpha-1-acid glycoprotein protects against renal ischemia-reperfusion injury by inhibition of inflammation and apoptosis. Transplantation.

[79] Etheridge A., Lee I., Hood L., Galas D., Wang K. (2011). Extracellular microRNA: A new source of biomarkers. Mutat. Res..

[80] Laterza O.F., Lim L., Garrett-Engele P.W., Vlasakova K., Muniappa N., Tanaka W.K., Johnson J.M., Sina J.F., Fare T.L., Sistare F.D. (2009). Plasma MicroRNAs as sensitive and specific biomarkers of tissue injury. Clin. Chem..

[81] Bailey W.J., Barnum J.E., Erdos Z., LaFranco-Scheuch L., Lane P., Vlasakova K., Sistare F.D., Glaab W.E. (2019). A Performance Evaluation of Liver and Skeletal Muscle-Specific miRNAs in Rat Plasma to Detect Drug-Induced Injury. Toxicol. Sci..

[82] Zhang C.-D., Lv F.-Q., Li Q.-Y., Zhang Y., Shi X.-Q., Li X.-Y., Tang J. (2014). Application of contrast-enhanced ultrasonography in the diagnosis of skeletal muscle crush injury in rabbits. Br. J. Radiol..

[83] Xu Q., Tian M., Xia J., Zhu W., Yang L. (2021). Application of Ultrasonography in the Diagnosis of Rhabdomyolysis. Ultrasound Med. Biol..

[84] Su B.-H., Qiu L., Fu P., Luo Y., Tao Y., Peng Y.-L. (2009). Ultrasonic appearance of rhabdomyolysis in patients with crush injury in the Wenchuan earthquake. Chin. Med. J..

[85] Zhao P., Li Q., Wang S., Wang Y., Zhu J., Zhu L., Tang J., Luo Y. (2022). Quantitative Analysis of Renal Perfusion in Rhabdomyolysis-Induced Acute Kidney Injury Using Contrast-Enhanced Ultrasound: An Experimental Study. Ultrasound Med. Biol..

[86] Lamminen A.E., Hekali P.E., Tiula E., Suramo I., Korhola O.A. (1989). Acute rhabdomyolysis: Evaluation with magnetic resonance imaging compared with computed tomography and ultrasonography. Br. J. Radiol..

[87] Russ P.D., Dillingham M. (1991). Demonstration of CT hyperdensity in patients with acute renal failure associated with rhabdomyolysis. J. Comput. Assist. Tomogr..

[88] Nakanishi K., Shimamoto S., Kishi M., Yoshioka T., Ishida T., Tomoda K., Nakamura H. (1997). CT, MR imaging and muscle biopsy in severe crush injury. Acta Radiol..

[89] Zhang L., Fang Z.-J., Liu F., Fu P., Tao Y., Li Z.-Y., Song B. (2011). Magnetic resonance imaging and magnetic resonance angiography in severe crush syndrome with consideration of fasciotomy or amputation: A novel diagnostic tool. Chin. Med. J..

[90] Shintani S., Shiigai T. (1993). Repeat MRI in acute rhabdomyolysis: Correlation with clinicopathological findings. J. Comput. Assist. Tomogr..

[91] Shuler M.S., Reisman W.M., Cole A.L., Whitesides T.E., Moore T.J. (2011). Near-infrared spectroscopy in acute compartment syndrome: Case report. Injury.

[92] Aoki N., Demsar J., Zupan B., Mozina M., Pretto E.A., Oda J., Tanaka H., Sugimoto K., Yoshioka T., Fukui T. (2007). Predictive model for estimating risk of crush syndrome: A data mining approach. J. Trauma.

[93] Zhang L., Fu P., Wang L., Cai G., Zhang L., Chen D., Guo D., Sun X., Chen F., Bi W. (2012). The clinical features and outcome of crush patients with acute kidney injury after the Wenchuan earthquake: Differences between elderly and younger adults. Injury.

[94] Najafi I., van Biesen W., Sharifi A., Hoseini M., Rashid Farokhi F., Sanadgol H., Vanholder R. (2008). Early detection of patients at high risk for acute kidney injury during disasters: Development of a scoring system based on the Bam earthquake experience. J. Nephrol..

[95] Paul A., John B., Pawar B., Sadiq S. (2009). Renal profile in patients with orthopaedic trauma: A prospective study. Acta Orthop. Belg..

[96] McMahon G.M., Zeng X., Waikar S.S. (2013). A risk prediction score for kidney failure or mortality in rhabdomyolysis. JAMA Intern. Med..

[97] Gao R., Zhao S., Aishanjiang K., Cai H., Wei T., Zhang Y., Liu Z., Zhou J., Han B., Wang J. (2021). Deep learning for differential diagnosis of malignant hepatic tumors based on multi-phase contrast-enhanced CT and clinical data. J. Hematol. Oncol..

[98] Byeon S.K., Madugundu A.K., Garapati K., Ramarajan M.G., Saraswat M., Kumar-M P., Hughes T., Shah R., Patnaik M.M., Chia N. (2022). Development of a multiomics model for identification of predictive biomarkers for COVID-19 severity: A retrospective cohort study Development of a multiomics model for identification of predictive biomarkers for COVID-19 severity: A retrospective cohort study. Lancet Digit. Health.

[99] Cheerla A., Gevaert O. (2019). Deep learning with multimodal representation for pancancer prognosis prediction. Bioinformatics.

